# The Condyle–Fossa Relationship After Maxillary Expansion with Tooth-Borne and Tooth-Bone-Borne Appliances: A Retrospective Cone-Beam Computed Tomography Study at the End of Retention

**DOI:** 10.3390/jcm15218503

**Published:** 2026-11-02

**Authors:** Esra Ağaoğlu Mogul, Merve Yelken Kendirci, Melda Pelin Akkitap

**Affiliations:** 1 Department of Orthodontics, Faculty of Dentistry, Biruni University, 34015 Istanbul, Türkiye; 2 Department of Dentomaxillofacial Radiology, Faculty of Dentistry, Biruni University, 34015 Istanbul, Türkiyemakkitap@biruni.edu.tr (M.P.A.)

**Keywords:** cone-beam computed tomography, mandibular condyle, orthodontic anchorage procedures, palatal expansion technique, temporomandibular joint

## Abstract

**Background/Objectives:** Maxillary expansion changes the occlusion and may alter the position of the mandibular condyle. Most cone-beam computed tomography (CBCT) studies have assessed the joint shortly after tooth-borne expansion and in the sagittal plane only. This study evaluated the condyle–fossa relationship from before expansion to the end of passive retention in the sagittal and coronal planes. **Methods:** In this retrospective study, 33 adolescents aged 12–17 years (66 condyles) treated with a tooth-borne Hyrax appliance (*n* = 16) or a tooth-bone-borne microimplant-assisted rapid palatal expander (MARPE; *n* = 17) were analysed. CBCT scans obtained before expansion (T0) and at the end of retention (T1; mean T0–T1 interval 6.3 months) were used to measure five joint spaces, the Pullinger concentricity index, intermolar width, overjet and overbite. Changes were assessed with paired tests and equivalence testing (±0.5 mm). **Results:** No joint space changed significantly (largest mean change 0.25 mm), and nine of ten were equivalent to no change within ±0.5 mm. The Pullinger index did not change, although individual category changes were frequent. Intermolar width increased by 5.07 mm (*p* < 0.001). No difference between appliances was detected in exploratory, baseline-adjusted analyses. **Conclusions:** No systematic group-level change in the condyle–fossa relationship was detected at the end of retention; individual variability was considerable.

## 1. Introduction

Maxillary transverse deficiency is a common skeletal discrepancy in growing patients, and rapid maxillary expansion (RME) is a well-established orthopaedic approach for its correction [1]. Conventional tooth-borne expanders transmit the expansion force through the posterior teeth, while microimplant-assisted rapid palatal expansion (MARPE) transfers part of that force directly to the palatal bone; both are in routine clinical use, and the choice between them usually follows the patient’s skeletal maturity [2]. A randomised comparison of the two found greater skeletal widening and less buccal displacement of the anchor teeth with the microimplant-assisted appliance [3].

Tooth-borne expanders are simple and effective in growing patients [4], but part of the expansion is dentoalveolar, with buccal tipping of the anchor teeth and thinning of the buccal alveolar bone [2,5]. Slow expansion with appliances such as the quad-helix produces mainly dentoalveolar changes and the greatest buccolingual tipping among commonly used appliances [6]. Tooth-bone-borne and bone-borne appliances produce a greater skeletal component and less tipping of the anchor teeth, and extend non-surgical expansion into late adolescence [2,3,5]; their disadvantages include miniscrew loosening, failure of sutural opening in some patients, and dentoalveolar and periodontal side effects that still require monitoring [7]. In skeletally mature patients, surgically assisted expansion remains an option, but it is invasive and is also associated with marked buccal tipping of the posterior teeth [8,9]. Because these appliances differ in force distribution and in the ratio of skeletal to dental expansion, their effects on the occlusion, and potentially on the temporomandibular joint, may also differ.

The effects of expansion may extend beyond the maxilla. Haas described it as the beginning rather than the end of dentofacial orthopaedics, noting that the forces reach the structures surrounding the maxilla [1]. Posterior teeth tip buccally, occlusal contacts change, and the mandible may rotate, all of which have been proposed to influence the relationship between the mandibular condyle and the glenoid fossa [5,10]. The evidence, however, is inconsistent. A systematic review of eight studies found that RME can modify the condyle–fossa relationship in the short term while the symmetry between the two condyles is maintained or improved, though it graded the certainty of that evidence as low to very low [11]. Individual studies have reported alterations in condylar position and joint spaces [12,13], limited or transient changes [14], and no change at all [10,15]. Patient characteristics and the timing of the post-expansion assessment differ widely across these reports, which makes them difficult to compare.

Timing is the clearest of these differences. Many studies measured the joint immediately or shortly after active expansion, while occlusal interferences were still present [12,13]. None of the eight studies in the review above examined the joint beyond the retention period, and the authors asked for longer follow-up covering post-retention stability [11]. A later report comparing tooth-borne with tooth-bone-borne appliances measured patients three months after expansion and concluded with the same request [10]. Most investigations have also confined themselves to the sagittal plane. Since expansion acts mainly in the transverse dimension, displacement of the condyle would be expected to appear medially or laterally, which a sagittal section cannot show.

This retrospective study therefore evaluated changes in the condyle–fossa relationship from pre-expansion to the end of passive retention in adolescents treated with maxillary expansion. Its primary objective was to determine whether the condyle–fossa relationship changed over the observation period in the overall cohort. Appliance type was not allocated but followed clinical judgement, and was therefore examined only as a secondary, exploratory factor. The hypothesis was that the condyle–fossa relationship would remain stable between pre-expansion and the end of retention.

## 2. Materials and Methods

### 2.1. Study Design and Ethical Approval

The institutional archive was screened for patients who had undergone maxillary expansion as part of routine orthodontic care. The study was carried out by the Departments of Orthodontics and Oral and Maxillofacial Radiology, Faculty of Dentistry, Biruni University, Istanbul, Türkiye. Cone-beam computed tomography (CBCT) was performed before treatment for diagnosis and appliance planning. The second examination was not acquired automatically; in each patient, it was requested individually by the treating orthodontist on the day that the expander was removed to assess the outcome of expansion, including mid-palatal sutural opening and the skeletal and dentoalveolar expansion achieved [2,3]; to evaluate the anchor teeth for the known dentoalveolar effects of expansion (buccal tipping, thinning of the buccal bone, dehiscence or fenestration, and root resorption) [7,16]; to assess the upper airway [17]; and to plan root positions and alveolar limits for the fixed-appliance phase. Every scan analysed here therefore already existed. No scan was taken for the study, and no patient received additional radiation or any other intervention because of it.

The study was approved by the Scientific Research Ethics Committee of Biruni University on 27 April 2026 (approval no. 2024-BİAEK/20-43) and followed the Declaration of Helsinki. The ethics committee waived the requirement for individual informed consent, given the retrospective design and the use of anonymised records. At the start of treatment, the parents or legal guardians of all patients had given general written informed consent for diagnostic records to be used for scientific and educational purposes, including publication.

### 2.2. Participants

Patients were included if they (1) had maxillary transverse deficiency without functional mandibular shift and had been treated with a Hyrax or a microimplant-assisted expander, (2) were aged 12 to 17 years at the start of treatment, and (3) had CBCT records available before expansion (T0) and at the end of retention (T1). The indication for expansion and the absence of a functional shift were established by clinical examination before treatment and retrieved from the patients’ records.

Patients were excluded if they had received previous orthodontic treatment; had a systemic or genetic disease, a craniofacial anomaly, or a history of temporomandibular joint disease, surgery or trauma; did not complete the expansion and retention protocol; lost a microimplant during expansion; or had scans with motion artefacts or in which either temporomandibular joint did not fall completely within the field of view [10,18]. The upper age limit was set to keep the sample within the adolescent range and to reduce age-related heterogeneity; it also matches the range used in earlier CBCT studies of condylar position after rapid maxillary expansion [15].

The archive was screened for patients treated with maxillary expansion during 2024. The pre-expansion scans of the included patients were obtained between February and December 2024, and their end-of-retention scans between July 2024 and July 2025. The search identified 38 patients treated with a Hyrax or microimplant-assisted expander, all of whom were assessed for eligibility. Five were excluded: two because they were 18 years or older at the start of treatment (21 and 23 years), one because treatment was discontinued, one because of microimplant loss during expansion, and one because a temporomandibular joint was partly outside the field of view. The remaining 33 patients (16 males, 17 females; mean age 15.0 ± 1.5 years, range 12.1 to 17.3) were included, giving 66 condyles for analysis. The mean interval between the two scans was 6.3 ± 0.6 months (range 4.7 to 7.3). The T1 scan was obtained on the day that the expander was removed, after its removal and before fixed appliances were placed, so that neither scan contained the expansion appliance. The date on which active expansion ended was not recorded consistently, so the passive retention period could not be reported separately from the total T0–T1 interval. Because every eligible patient treated during the study period was included, no sample size was calculated in advance. The sample size is comparable to that of previous CBCT studies of condylar changes after expansion [10,14,18,19].

### 2.3. Expansion Protocols

All patients were treated as part of routine care, before and independently of this study. The choice of appliance depended on the clinical assessment of skeletal maturity [2].

**Figure 1:**
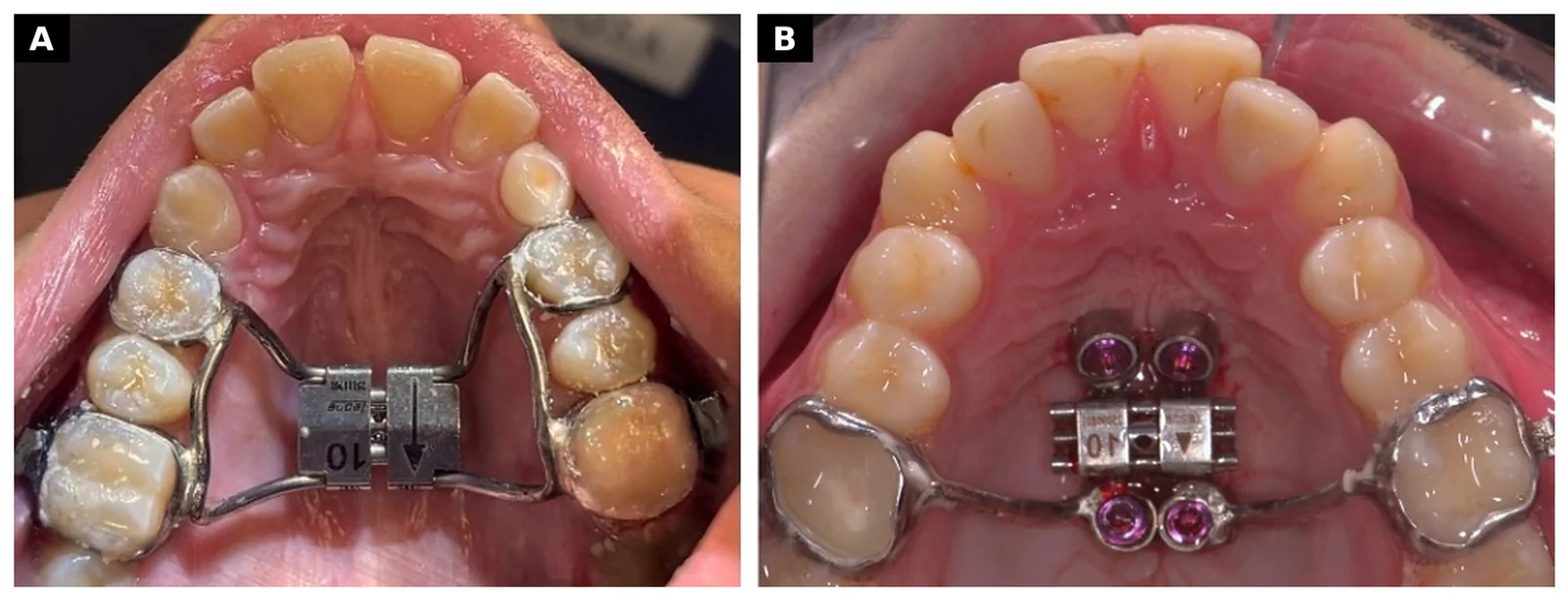
Expansion appliances used in study. (**A**) Conventional tooth-borne Hyrax appliance banded to maxillary first premolars and first molars. (**B**) Customised tooth-bone-borne appliance banded to maxillary first molars and supported by four paramedian palatal microimplants, shown immediately after insertion.

Sixteen patients were treated with a conventional tooth-borne Hyrax appliance banded to the maxillary first premolars and first molars (Figure 1A). The other 17 received a customised tooth-bone-borne appliance (Figure 1B), banded to the maxillary first molars and supported by four paramedian palatal microimplants (Guide-Lock, Fast Drive Anchor, İzmir, Türkiye), each 2.2 mm in diameter and with an intraosseous length of at least 9 mm. For these patients, the pretreatment CBCT scan was sent to the manufacturer, where the appliance and the microimplant positions were planned on a digital model created by superimposing the scan data on the radiographic data, and the appliance was produced individually; implant length and position were therefore determined patient by patient. Both appliances were anchored to the maxillary first molars; they differed in the additional premolar bands of the Hyrax appliance and the palatal microimplants of the other. Both groups used a Leone expansion screw (Leone S.p.A., Sesto Fiorentino, Italy), the size being selected for each patient. One quarter-turn produced approximately 0.20 mm of expansion. The screw was turned twice a day during the first week and once a day afterwards, until the palatal cusps of the maxillary first molars met the buccal cusps of the mandibular first molars [19]. It was then locked, and the appliance stayed in place as a passive retainer until the second CBCT scan.

### 2.4. CBCT Acquisition

All scans came from one unit (CS 9600, Carestream Dental LLC, Atlanta, GA, USA) with a standardised protocol: a 16 × 17 cm field of view, a 150 µm voxel size, 120 kV, 5.0 mA, a 0.7 mm copper filter and a 40 s acquisition time. The dose area product was 2654 mGy·cm^2^ per scan. A field of view of this size was selected so that the dentoalveolar region, both temporomandibular joints and the airway could be evaluated in a single examination; comparable large-volume protocols have been used in CBCT studies of condylar changes after expansion [10,18]. Lower-dose two-dimensional radiographs were not adequate for these clinical questions, because panoramic and cephalometric radiographs cannot show mid-palatal sutural opening in three dimensions, the buccal cortical bone or the bucco-palatal position of the roots. The CBCT examination replaced these radiographs rather than being added to them: no separate panoramic or cephalometric radiograph was taken at T1. Patients were scanned in a standing position, in maximum intercuspation, using the manufacturer’s standard head-positioning supports. Digital Imaging and Communications in Medicine (DICOM) data were analysed with CS Imaging software, version 8.0.25 (Carestream Dental LLC, Atlanta, GA, USA). CBCT provides reliable measurements of temporomandibular joint spaces [20].

### 2.5. Joint Space Measurements

**Figure 2:**
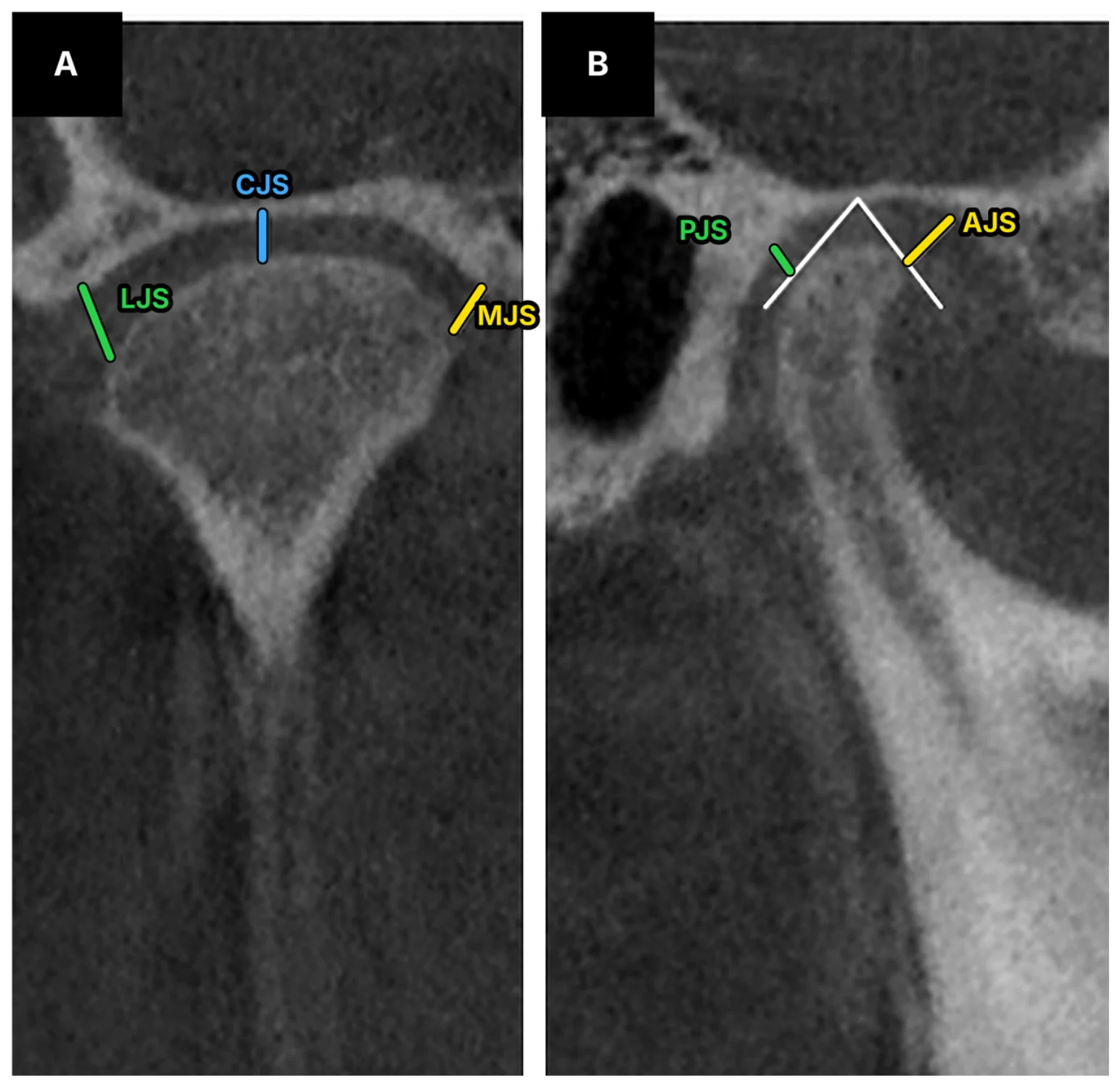
Joint space measurements on oblique CBCT sections. (**A**) The oblique coronal section: LJS (green line), CJS (blue line) and MJS (yellow line). (**B**) The oblique sagittal section: PJS (green line) and AJS (yellow line); white lines indicate the tangents drawn from the most superior point of the glenoid fossa.

CBCT volumes were first reoriented with the Frankfort horizontal plane parallel to the axial plane and the midsagittal plane perpendicular to it. The right and left temporomandibular joints were then evaluated separately. On the axial section showing the greatest mediolateral dimension of the condylar head, the long axis of that condyle was identified, and oblique coronal and sagittal sections were reconstructed parallel and perpendicular to this axis. Because the long axes of the two condyles are not parallel, the sections were defined independently for each joint [20,21]. Measurements were made on 150 µm slices at 1.48× magnification (Figure 2).

Joint spaces were recorded as the shortest distance between the corresponding points on the condyle and the glenoid fossa. On the oblique sagittal section, the anterior joint space (AJS) and posterior joint space (PJS) were measured between the most anterior and most posterior points of the condyle and the corresponding points of the fossa [22]. On the oblique coronal section, the medial (MJS) and lateral (LJS) joint spaces were measured from the medial and lateral poles of the condyle to the corresponding walls of the fossa, and the central joint space (CJS) between the central surface of the condyle and the corresponding surface of the fossa [23].

Sagittal condylar position was described with the concentricity index of Pullinger and Hollender [24], calculated as (PJS − AJS)/(PJS + AJS) × 100. Values between −12% and +12% were read as concentric, lower values as a posterior condyle and higher values as an anterior one; this threshold corresponds to the displacement at which a shift first becomes perceptible on subjective evaluation [25] and has been applied in the same way to CBCT records of adolescents [21]. For the transverse plane, a corresponding coronal index, (LJS − MJS)/(LJS + MJS) × 100, was calculated and treated as exploratory.

### 2.6. Dental Measurements

Maxillary intermolar width was measured on the axial section at the level of the occlusal surfaces of the maxillary first molars as the distance between the central fossae of the right and left first molars [14,26]. Overjet and overbite were also measured on the CBCT data at both time points.

### 2.7. Measurement Procedure and Repeatability

All measurements were made by two assistant professors of oral and maxillofacial radiology, working together and agreeing on each value (consensus measurement). Four weeks later, they repeated the procedure, without access to the first set of measurements, on the T0 and T1 scans of 10 consecutive patients (20 scans, 40 condyles) whose pre-expansion scans had been obtained between June and August 2024. Because both sessions used the same consensus procedure, the analysis quantifies the repeatability of the consensus measurement procedure; independent inter-examiner agreement was not assessed.

The repeatability between the two sessions was expressed as intraclass correlation coefficients (ICCs; two-way random effects, absolute agreement, single measurement) [27] with F-based 95% confidence intervals [28]. Bland–Altman analysis gave the mean bias with 95% limits of agreement [29]. The standard error of measurement (SEM) was calculated as the standard deviation of the between-session differences divided by √2, separately for each measurement, side and time point, from the paired session values of the 10 patients; each patient contributed one pair of measurements to each estimate. Because a change from T0 to T1 combines the measurement errors of two different scans, the smallest detectable change (SDC) for an individual patient’s change was calculated as 1.96 × √(SEM^2^ at T0 + SEM^2^ at T1) and was used to judge whether an observed change exceeded measurement error [30]. For the Pullinger index, a single SEM was pooled across sides and time points (root mean square of between-session differences divided by √2), reducing its SDC to 1.96 × √2 × SEM. The corresponding error of a group mean change was obtained as SDC/√*n* with *n* = 33, the number of independent patients in each side-specific analysis; the two condyles of a patient were not treated as independent observations.

### 2.8. Statistical Analysis

All analyses were run in Python 3 (pandas, SciPy 1.17, statsmodels), with two-sided tests and α = 0.05. Continuous variables are reported as the mean ± standard deviation (SD) and categorical variables as *n* (%). The normality of the T1 − T0 difference variables was assessed with the Shapiro–Wilk test together with skewness and kurtosis; paired t tests were used where normality held and Wilcoxon signed-rank tests where it did not. The two appliance groups were compared for age and inter-scan interval with the independent-samples t test (Welch’s correction where variances were unequal) and for sex with Fisher’s exact test, and their baseline values with the Mann–Whitney U test.

The primary analysis was the change in each joint space from T0 to T1 in the whole sample, reported for each side with the mean change, 95% confidence interval (CI) and paired Cohen’s dz. The Benjamini–Hochberg procedure controlled the false discovery rate (FDR) within each family of tests [31]. Because a non-significant result does not by itself establish the absence of change, each joint space was also tested for equivalence with two one-sided tests (TOSTs) against a margin of ±0.5 mm [32]. No validated threshold exists for a clinically meaningful change in CBCT-derived joint spaces. The margin was therefore set at ±0.5 mm because it corresponds to approximately three voxels at the 150 µm resolution used, lies well above the measurement error of a group mean change in this study (Section 3.2), and is of the same order as the largest mean joint space changes reported after expansion [14,33]. The margin applies to mean changes; individual changes were interpreted against the SDC. The proportion of patients whose change exceeded 0.5, 1.0 and 1.5 mm was tabulated. All primary comparisons were repeated with 10,000 bootstrap resamples and 10,000 sign permutations.

Because each patient contributed two condyles, the results were confirmed in linear mixed-effects models of the form measurement ~ time + (1|patient) + (1|patient:side), which analyse both sides together while accounting for within-patient dependence; the time × side interaction was tested by means of a likelihood-ratio test. The same models were applied to the Pullinger and coronal indices. Transitions between Pullinger categories were examined for each side with the McNemar–Bowker test [34] and weighted kappa [35], and right-left symmetry was compared at each time point.

Changes in intermolar width, overjet and overbite were tested in the same way. Appliance type was not randomly allocated; it followed skeletal maturity. It was therefore examined only as a secondary, exploratory factor, in analysis of covariance models of the form change ~ appliance + baseline value + age + inter-scan interval; adjustment for the baseline value of each outcome accounts for baseline differences between the groups and for regression to the mean. These associations are reported without causal interpretation. The relationship between the changes in intermolar width, overjet and overbite and the changes in joint spaces was explored with Pearson correlation.

## 3. Results

### 3.1. Sample Characteristics

A total of 33 patients met the inclusion criteria, giving 66 condyles for analysis. Sixteen were treated with the tooth-borne Hyrax appliance and seventeen with the MARPE appliance. The mean interval between the two scans was 6.26 ± 0.64 months and did not differ between the groups (*p* = 0.211). Sex distribution was also comparable (*p* = 0.494). The MARPE group was older at T0 than the Hyrax group (15.74 ± 1.27 versus 14.20 ± 1.35 years; *p* = 0.002; Cohen’s d = −1.18), consistent with the clinical rule used to select the appliance (Table 1).

At baseline, the two groups were comparable for 15 of the 17 variables examined. Two differences survived correction for multiple testing: the right medial joint space was wider in the MARPE group (3.35 ± 0.94 versus 2.17 ± 0.68 mm; *p*_FDR = 0.011) and the right coronal index differed between groups (*p*_FDR = 0.038). No baseline difference was found for any other joint space, for the Pullinger index, or for intermolar width (Table S1, Supplementary Materials).

### 3.2. Measurement Repeatability

The repeatability of the consensus measurement procedure was high for every variable. Intraclass correlation coefficients ranged from 0.860 to 0.996, with the lowest value for the left anterior joint space at T1 and the highest for overjet. Mean bias was below 0.16 mm throughout. For the joint spaces, the SEM ranged from 0.05 to 0.23 mm and the SDC for a T0–T1 change from 0.28 to 0.46 mm; the 95% limits of agreement exceeded ±0.5 mm for four of the twenty joint space measurements, all at T1 (maximum 0.75 mm, left medial joint space). The corresponding error of a group mean change (SDC/√33) was 0.05 to 0.08 mm. Intermolar width had the widest limits (−2.21 to 1.97 mm at T0), reflecting the larger absolute magnitude of that measurement (Table 2).

### 3.3. Changes in Joint Spaces

No joint space changed significantly between T0 and T1 on either side (Table 3). The largest mean change was an increase of 0.248 mm in the left posterior joint space (95% CI −0.062 to 0.559; *p* = 0.087), and the smallest was an increase of 0.018 mm in the right central joint space (*p* = 0.907). All paired effect sizes (Cohen’s dz) were small, |dz| ≤ 0.28, and no comparison remained significant after correction for multiple testing (all *p*_FDR ≥ 0.558). Bootstrap confidence intervals and sign-permutation tests gave the same conclusions (Table S8).

**Figure 3:**
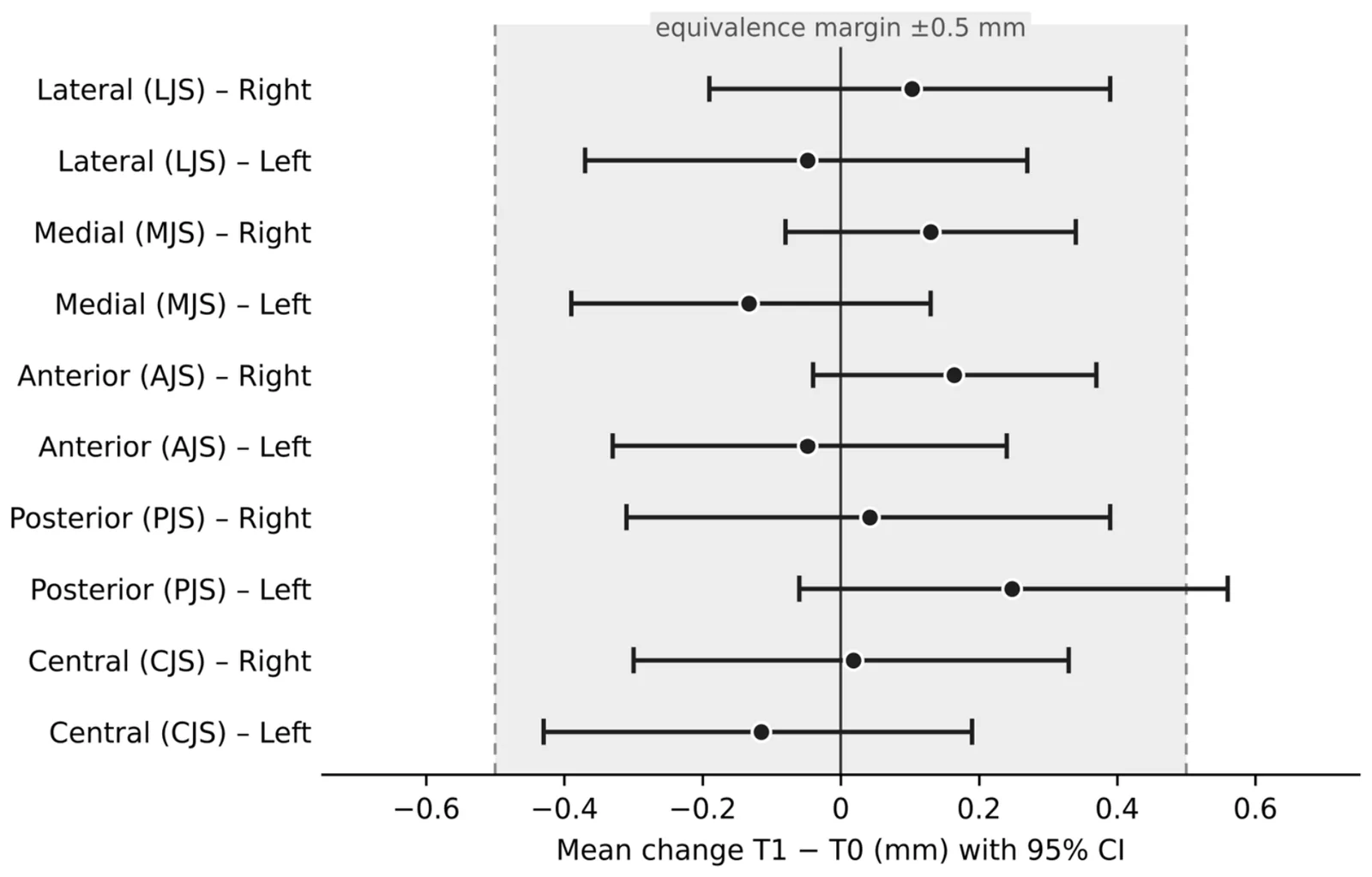
The mean change in each joint space with 95% confidence intervals. The shaded band marks the ±0.5 mm equivalence region.

Equivalence testing supported these findings rather than merely failing to reject the null hypothesis. Nine of the ten joint spaces were statistically equivalent to no change within a margin of ±0.5 mm (all *p* ≤ 0.008). The exception was the left posterior joint space, for which equivalence was not established (*p* = 0.054), its confidence interval reaching the margin. The equivalence analysis is summarised in Table 4 and Figure 3.

At the level of individual patients, changes were more variable. Between 33% and 58% of patients showed a change greater than 0.5 mm in a given joint space, 6% to 24% exceeded 1.0 mm, and 0% to 15% exceeded 1.5 mm (Table S2). These changes were bidirectional and cancelled out at the group level. Because the SDC for an individual T0–T1 change was 0.28 to 0.46 mm (Table 2), every change greater than 0.5 mm exceeded measurement error.

The mixed-effects models, which analysed both sides together while accounting for within-patient dependence, confirmed the absence of any systematic change (Table S3). Time effects ranged from −0.048 to +0.145 mm, with none approaching significance (all *p* ≥ 0.204; all *p*_FDR = 0.985), and no time × side interaction was significant (*p* ≥ 0.105). The between-patient intraclass correlation ranged from 0.35 to 0.61 for the joint spaces, indicating moderate similarity between the two sides within a patient. Right and left joint spaces did not differ significantly at either time point (Table S9).

### 3.4. Condylar Position Indices

Neither the Pullinger index nor the coronal index changed significantly from T0 to T1 (Table S4). Changes ranged from −3.39 to +4.22 percentage points, with confidence intervals spanning zero in every case and effect sizes of |dz| ≤ 0.21.

**Figure 4:**
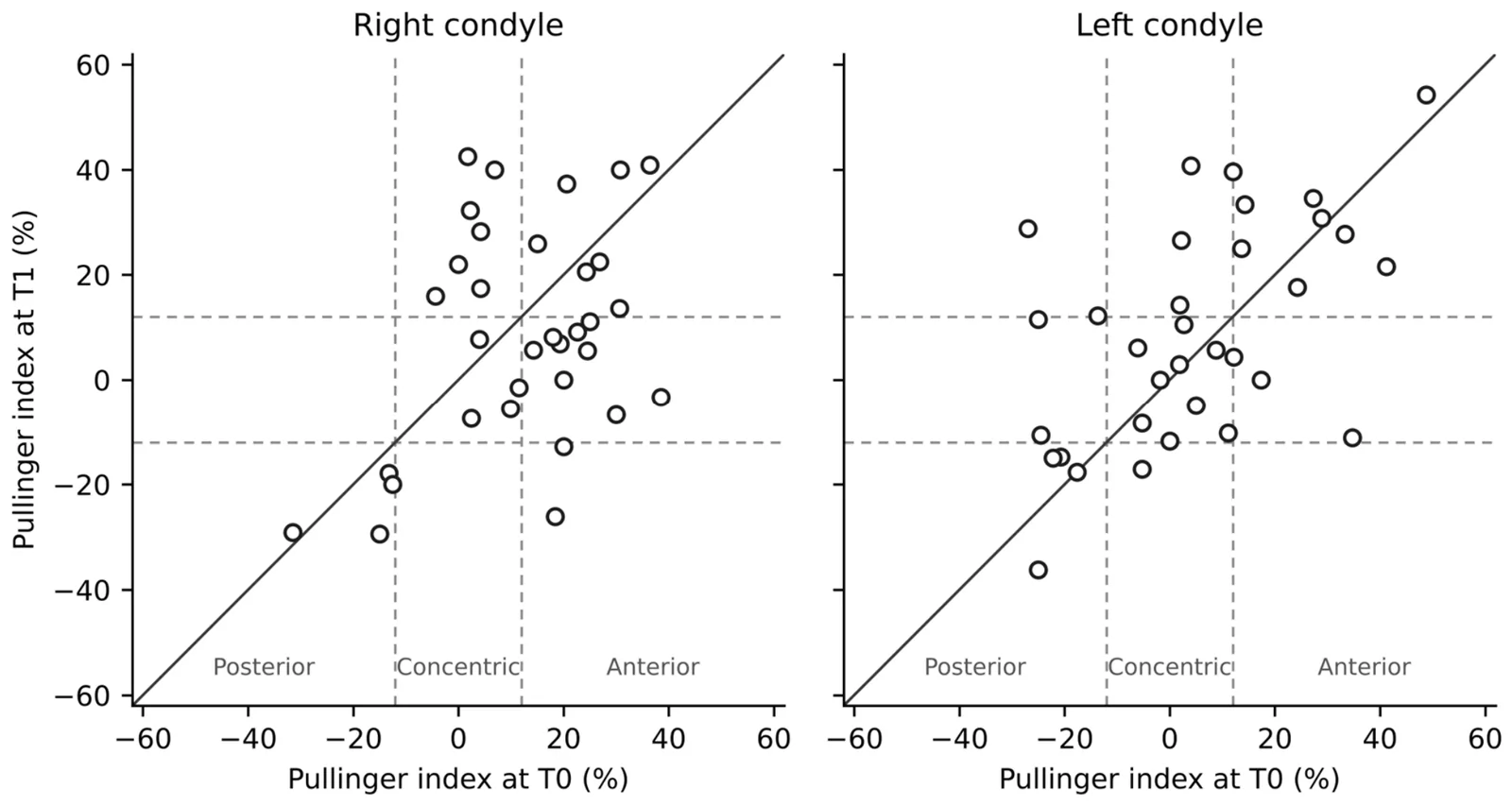
The Pullinger index at T0 and T1. Dashed lines mark the ±12 category boundaries and the solid line marks the line of no change.

The distribution of Pullinger categories was essentially unchanged. On the right side, 4 condyles were posterior, 11 concentric and 18 anterior at T0, compared with 6, 13 and 14 at T1; on the left, the corresponding figures were 8, 14 and 11 at T0 and 5, 14 and 14 at T1. Transitions were symmetric on both sides (McNemar–Bowker *p* = 0.325 right, *p* = 0.480 left), with no directional shift. However, the category remained unchanged in only 45.5% of right and 63.6% of left condyles; thus, agreement between T0 and T1 was fair on the right and moderate on the left (weighted κ = 0.21 and 0.47). Agreement between the two measurement sessions, by contrast, was almost perfect (weighted κ = 0.97), and the smallest detectable change for the index was 11.85 percentage points, about half the width of the concentric category (Table 5, Figure 4).

### 3.5. Dental Changes

All three dental measurements changed significantly (Table 6). Maxillary intermolar width increased by 5.07 mm (95% CI 4.21 to 5.93; *p* < 0.001; dz = 2.09), overjet increased by 1.40 mm (95% CI 0.61 to 2.19; *p* = 0.001; dz = 0.63), and overbite decreased by 1.38 mm (95% CI −2.16 to −0.60; *p* = 0.001; dz = −0.63). Bootstrap confidence intervals agreed with the parametric intervals.

### 3.6. Exploratory Analyses

No association between dental change and joint space change survived correction for multiple testing (Table S5). No correlation reached nominal significance even before correction (all |*r*| ≤ 0.28; all *p* ≥ 0.114).

In the exploratory comparison, no difference between appliances was detected in the change in any joint space or index after adjustment for the baseline value, age and inter-scan interval (Table S6). Adjusted differences between MARPE and Hyrax ranged from −0.087 to +0.370 mm for the joint spaces, with all confidence intervals spanning zero (all *p* ≥ 0.199; all *p*_FDR = 0.946). Without adjustment for the baseline value, the largest apparent difference was in the right coronal index (+12.2 percentage points; *p* = 0.088); after adjustment for its baseline imbalance, the difference decreased to +5.6 percentage points (95% CI −9.4 to 20.6; *p* = 0.450).

For the dental measurements, the increase in intermolar width did not differ between the Hyrax and MARPE groups (+5.25 versus +4.89 mm; adjusted difference −1.25 mm, 95% CI −3.12 to 0.63; *p* = 0.185), and neither did the changes in overjet and overbite (Table S7).

## 4. Discussion

This study found no systematic group-level change in the condyle–fossa relationship between pre-expansion and the end of passive retention, even though the maxilla had widened by about 5 mm at the first molars. Individual joints did change, in some patients by more than 0.5 mm, but the changes ran in both directions.

These findings agree with the studies closest in design to this one. Eglenen et al. compared tooth-borne and tooth-bone-borne appliances in 25 adolescents and found no change in condylar position three months after expansion in either group [10]. Qi et al. followed 20 adults (mean age 21.1 years) treated with a microimplant-supported expander through six months of retention and found that the coronal joint spaces remained unchanged throughout [14]. McLeod et al., who included an untreated control group and the same six-month interval, reported small condylar positional changes in both the expansion and the control group, with no difference between them [15]. Mehta et al. compared bone-anchored with tooth-anchored expansion over the long term and found no difference in the condyle–fossa relationship between techniques [33].

One finding does not align. Qi et al. reported that the superior joint space, measured in the sagittal plane, remained slightly increased after six months of retention (+0.21 mm), while the posterior space had returned to baseline. This study assessed the vertical dimension of the joint in the coronal plane, as the central joint space, which did not change. A sagittal superior joint space was not measured here, so a small persistent change in that particular dimension cannot be excluded. More generally, mean changes smaller than the ±0.5 mm equivalence margin, such as the +0.21 mm reported by Qi et al., cannot be excluded with these data.

The studies that did report condylar displacement after expansion differ from the present study in two ways that matter. The first is the measurement time point. Melgaço et al. scanned three weeks after expansion and Leonardi et al. at the end of an activation period averaging 18 days [12,13]. Chen et al. scanned adults immediately after microimplant-assisted expansion and found that the condyle had moved inferiorly by 0.96 mm on the deviated side and 0.70 mm on the non-deviated side, although the relationship between the two sides was unchanged [19]. At that point, the posterior teeth have just been separated, occlusal interferences are present, and the mandible closes into a position that will change. Eglenen et al. argued that the occlusion settles during retention and the condyles return to where they started, which would explain why longer studies find nothing. These data fit that reading, although they cannot show it directly, because no scan was obtained at the end of active expansion.

The second difference relates to the patients. Many of the studies reporting change examined children with functional unilateral crossbite, in whom the mandible shifts on closure and the two condyles sit asymmetrically [13,36]. It has been argued that the condyle on the crossbite side is the more normally positioned of the two, and that expansion corrects the position of the contralateral condyle rather than displacing a normal one; on this reading, if condylar position is normal before expansion, no change is to be expected [10]. What is measured in those studies is then the loss of an existing displacement, not an effect of expansion on a normally seated joint. None of the patients in this study had a functional shift, so there was no displacement to correct, and none appeared. The result here fits that prediction.

Skeletal maturity may also contribute. Wang et al. reported that condylar position changes less after expansion in patients with more advanced skeletal maturation [18]. This sample averaged 15.0 years, and the MARPE group 15.7 years, which places most patients past the peak growth period.

A negative result means little unless the measurement is precise enough to detect a change. The consensus measurement procedure was highly repeatable (ICC 0.86–0.996). The 95% limits of agreement, which describe the error of a single measurement, exceeded ±0.5 mm for four joint space measurements at T1, and the SDC for an individual T0–T1 change was 0.28 to 0.46 mm; individual changes greater than 0.5 mm therefore exceed measurement error, although the SDC does not include differences in mandibular position between the two scans [30]. The error of a group mean change was much smaller (0.05–0.08 mm), so the absence of a systematic change in the cohort is not explained by measurement imprecision.

Equivalence testing addresses the same question directly [32]. Nine of the ten joint spaces were statistically equivalent to no change within ±0.5 mm. The margin reflects measurement resolution and the magnitude of changes reported in comparable studies rather than an established clinical threshold, which does not exist for joint spaces, and the equivalence findings should be interpreted in that context. The exception was the left posterior joint space, where the confidence interval reached the margin (*p* = 0.054); its mean change of 0.248 mm was the largest recorded and the only one approaching significance before correction.

The high repeatability probably reflects the 150 µm voxel size, the use of a single scanner throughout, the absence of the expansion appliance from both scans, and measurement by consensus between two observers. Consensus reduces the random variation between sessions, but it cannot reveal a systematic bias shared by both observers, and it leaves inter-observer variability unmeasured.

Group means were stable, but individual condyles were not. Between one-third and half of the patients showed a change greater than 0.5 mm in a given joint space, and a smaller number exceeded one millimetre. These changes occurred in both directions and largely cancelled out at the group level, a pattern more consistent with measurement and physiological variability than with a uniform directional treatment effect.

The Pullinger categories behaved the same way. The distribution across posterior, concentric and anterior positions was almost identical at the two time points and the transitions were symmetric on both sides, yet the category changed in more than half of right condyles, meaning that agreement between T0 and T1 was only fair on that side (weighted κ = 0.21). This is not a reproducibility failure of the index: agreement between the two measurement sessions was almost perfect (weighted κ = 0.97). The index is a ratio of two small measurements, so the errors of both are combined in it; its smallest detectable change was 11.85 percentage points, about half the width of the concentric category (−12% to +12%), so a condyle near a category boundary can change category through measurement error alone. Individual category shifts should therefore not be read as condylar movement. Ren et al. found that among asymptomatic volunteers with normally positioned discs, the condyle was central in 41%, posterior in 32% and anterior in 27% [37], close to the baseline spread in this sample. Condylar position is variable in healthy joints, and a classification with fixed cut-offs will move with it.

The coronal joint spaces in this sample were ordered differently from those reported by Ikeda et al. and Han et al. [38,39], most likely because the medial and lateral spaces were measured here from the condylar poles [23] rather than at sextant junctions; values obtained with the two definitions should not be compared directly.

Linear joint spaces describe where the condyle sits, not what the bone is doing. Dumanli Gok et al. used bone scintigraphy during expansion and found a marked increase in metabolic activity at the condyle, which they read as remodelling [40]. Bone remodelling of that kind would not necessarily alter the linear distance between two cortical surfaces measured on CBCT. The result should therefore be read narrowly. Stable linear joint spaces do not rule out remodelling within the joint.

Structural findings of this kind should not be read as evidence on joint function. Current consensus does not support a direct causal link between occlusal change and temporomandibular disorder [41], and no functional assessment was made in this study. These results show only that, after the maxilla had been widened by approximately 5 mm and the occlusion had settled through retention, no systematic group-level change in the bony relationship between the condyle and fossa was detected. Neither appliance was associated with such a change in exploratory analyses.

The main limitation is the absence of an untreated control group. Serial CBCT scans of untreated adolescents are difficult to justify ethically, and delaying expansion for research purposes could compromise treatment [18], so no such group could be assembled from a retrospective archive. The patients were still growing during the six months of follow-up, and the effects of expansion cannot be separated from those of growth and remodelling. Joint spaces describe the relationship between two structures that remodel together, which limits the problem but does not remove it. The controlled data that exist point in the same direction: in adolescents of the same age range followed for the same six months, condylar positional changes were small in both treated and untreated groups and did not differ between them [15].

Appliance type was not allocated but followed clinical judgement, so the two groups differed in age, and they also differed at baseline in the right medial joint space and the right coronal index. The comparison between appliances is therefore exploratory and should not be read as a treatment comparison. It found no difference in any joint space after adjustment for baseline values, age and follow-up interval, which is reassuring but not conclusive. The dentoalveolar evidence is worth setting beside it. A randomised trial found that the two appliance types act differently on the teeth and the alveolar bone, the anchor teeth being less displaced buccally with the microimplant-assisted appliance [3]. Whatever difference exists at that level did not show up in the joint spaces here.

Only the anatomical relationship between the condyle and fossa was assessed, not temporomandibular joint function, pain or patient-reported outcomes. Whether the stable group-level joint spaces reported here correspond to an absence of symptoms cannot be determined from these data, and the two do not necessarily go together.

Further limitations apply. The superior joint space was assessed only in the coronal plane, which limits direct comparison with studies reporting the sagittal superior space. The observers could not be blinded to appliance type, since the appliances and microimplants are visible on the scans, although this affects the exploratory comparison rather than the primary analysis. Measurement by consensus also means that inter-observer variability could not be quantified, and a systematic bias shared by both observers would not have been detected. Scans were available only before expansion and at the end of retention, so changes in between cannot be described; a study with a scan at the end of active expansion would show whether the condyle moves and returns, as has been proposed, or never moves at all.

In addition, the date on which active expansion ended was not recorded consistently, so the duration of passive retention could not be reconstructed retrospectively; the T0–T1 interval includes both the active expansion and the retention phases and cannot be interpreted as the duration of passive retention, and any effect of the length of retention could not be examined. Because T1 preceded fixed-appliance treatment, condylar adaptation occurring later [18] would not have been captured. The skeletal and dentoalveolar components of expansion, such as sutural opening, molar inclination and extrusion, were not measured, so the condylar findings could not be related to the pattern of expansion. The repeat measurements were obtained in 10 consecutive rather than randomly selected patients. The measurement error estimates were derived from these 10 patients and are therefore themselves imprecise. Finally, a large field of view involves a higher radiation dose than a small-volume or two-dimensional examination; smaller fields of view or low-dose protocols could reduce exposure, and these findings should not be taken as a justification for acquiring CBCT solely to assess the temporomandibular joint after maxillary expansion.

Future studies should use prospective designs with randomised allocation of appliances and, where ethically acceptable, an untreated or delayed-treatment comparison group, with follow-up beyond fixed-appliance treatment and an additional scan at the end of active expansion. Combining CBCT with magnetic resonance imaging of the articular disc and standardised clinical assessment of temporomandibular joint function would show whether radiographic changes have clinical relevance. Independent measurements by two examiners, together with measurement of sutural opening, molar inclination and extrusion, would allow condylar changes to be related to the pattern of expansion.

## 5. Conclusions

No systematic group-level change in the condyle–fossa relationship was detected between pre-expansion and the end of passive retention in adolescents treated with tooth-borne or tooth-bone-borne maxillary expansion, despite a transverse gain of approximately 5 mm at the first molars. Individual changes, including shifts in Pullinger category, were common and should be interpreted against measurement error. Differences between appliance types could be assessed only at an exploratory level, and changes during active expansion remain to be investigated.

## Figures and Tables

**Table 1:** Demographic characteristics and observation interval by group.

Variable	Hyrax (*n* = 16)	MARPE (*n* = 17)	Difference (95% CI)	Test	*p*
Age (years)	14.20 ± 1.35	15.74 ± 1.27	−1.54 (−2.47; −0.61)	Independent t	0.002
T0–T1 interval (months)	6.11 ± 0.81	6.40 ± 0.40	−0.29 (−0.73; +0.16)	Independent t	0.211
Sex (female/male)	7/9	10/7	—	Fisher’s exact	0.494

MARPE = microimplant-assisted rapid palatal expansion; SD = standard deviation; CI = confidence interval. Continuous variables are mean ± SD. Cohen d for age = −1.18. Sex was compared with Fisher’s exact test. Variances for T0–T1 interval were unequal (Levene *p* = 0.013), so Welch’s correction was applied. *p*-values are from independent-samples t tests (age, interval) or Fisher’s exact test (sex).

**Table 2:** Repeatability of consensus measurement procedure (10 patients, T0 and T1 scans).

Measurement	ICC (95% CI)	Bias (mm)	95% LoA (mm)	SEM (mm)	SDC (mm)
T0 (before expansion)					
Lateral (LJS)—Right	0.957 (0.845–0.989)	−0.080	−0.470 to 0.310	0.14	–
Medial (MJS)—Right	0.980 (0.927–0.995)	0.050	−0.199 to 0.299	0.09	–
Anterior (AJS)—Right	0.991 (0.963–0.998)	0.000	−0.244 to 0.244	0.09	–
Posterior (PJS)—Right	0.995 (0.979–0.999)	−0.010	−0.155 to 0.135	0.05	–
Central (CJS)—Right	0.992 (0.968–0.998)	0.000	−0.207 to 0.207	0.07	–
Lateral (LJS)—Left	0.993 (0.973–0.998)	0.020	−0.203 to 0.243	0.08	–
Medial (MJS)—Left	0.990 (0.960–0.997)	0.040	−0.125 to 0.205	0.06	–
Anterior (AJS)—Left	0.991 (0.966–0.998)	0.040	−0.190 to 0.270	0.08	–
Posterior (PJS)—Left	0.935 (0.774–0.983)	−0.060	−0.383 to 0.263	0.12	–
Central (CJS)—Left	0.988 (0.955–0.997)	−0.050	−0.299 to 0.199	0.09	–
Intermolar width	0.946 (0.802–0.986)	−0.120	−2.210 to 1.970	0.75	–
Overjet	0.996 (0.984–0.999)	−0.160	−0.700 to 0.380	0.20	–
Overbite	0.979 (0.919–0.995)	0.020	−0.884 to 0.924	0.33	–
T1 (end of retention)					
Lateral (LJS)—Right	0.989 (0.959–0.997)	0.060	−0.263 to 0.383	0.12	–
Medial (MJS)—Right	0.959 (0.847–0.990)	−0.020	−0.337 to 0.297	0.12	–
Anterior (AJS)—Right	0.981 (0.891–0.996)	0.130	−0.216 to 0.476	0.12	–
Posterior (PJS)—Right	0.988 (0.954–0.997)	−0.040	−0.412 to 0.332	0.13	–
Central (CJS)—Right	0.986 (0.947–0.996)	−0.070	−0.542 to 0.402	0.17	–
Lateral (LJS)—Left	0.962 (0.854–0.990)	0.010	−0.397 to 0.417	0.15	–
Medial (MJS)—Left	0.908 (0.693–0.976)	−0.120	−0.752 to 0.512	0.23	–
Anterior (AJS)—Left	0.860 (0.536–0.963)	0.040	−0.574 to 0.654	0.22	–
Posterior (PJS)—Left	0.941 (0.789–0.985)	0.050	−0.483 to 0.583	0.19	–
Central (CJS)—Left	0.980 (0.927–0.995)	−0.050	−0.486 to 0.386	0.16	–
Intermolar width	0.951 (0.815–0.988)	0.020	−1.663 to 1.703	0.61	–
Overjet	0.994 (0.976–0.998)	−0.060	−0.533 to 0.413	0.17	–
Overbite	0.986 (0.943–0.996)	−0.020	−0.617 to 0.577	0.22	–
T0 to T1 change (smallest detectable change)					
Lateral (LJS)—Right	–	–	–	–	0.36
Medial (MJS)—Right	–	–	–	–	0.29
Anterior (AJS)—Right	–	–	–	–	0.30
Posterior (PJS)—Right	–	–	–	–	0.28
Central (CJS)—Right	–	–	–	–	0.36
Lateral (LJS)—Left	–	–	–	–	0.33
Medial (MJS)—Left	–	–	–	–	0.46
Anterior (AJS)—Left	–	–	–	–	0.46
Posterior (PJS)—Left	–	–	–	–	0.44
Central (CJS)—Left	–	–	–	–	0.35
Intermolar width	–	–	–	–	1.90
Overjet	–	–	–	–	0.51
Overbite	–	–	–	–	0.77

ICC = intraclass correlation coefficient, for two-way random effects, absolute agreement, single measurement (ICC 2,1), with F-based 95% confidence intervals; bias = mean difference between sessions (second − first); LoA = 95% limits of agreement; CI = confidence interval; SEM = standard error of measurement; SDC = smallest detectable change for an individual patient’s T0–T1 change, 1.96 × √(SEM^2^ at T0 + SEM^2^ at T1); LJS, MJS, AJS, PJS, CJS = lateral, medial, anterior, posterior and central joint spaces. Measurements were repeated on the T0 and T1 scans of the same 10 consecutive patients. In the original submission, the T0 right LJS value of one patient had been entered with a decimal comma (2,4 instead of 2.4), was not recognised as a number by the statistical software and was excluded from that analysis (*n* = 9). The entry was corrected to the value recorded at the measurement session; it was neither re-measured nor imputed. All estimates are therefore based on the same 10 patients, and the sample size column was removed.

**Table 3:** Change in joint spaces from T0 to T1 (whole sample, *n* = 33).

Joint Space	T0 (mm)	T1 (mm)	Change (mm)	95% CI	*p*	*p* (FDR)	dz
Lateral (LJS)—Right	3.10 ± 1.23	3.20 ± 1.35	+0.103	(−0.187; 0.393)	0.474	0.790	0.13
Lateral (LJS)—Left	3.06 ± 1.12	3.01 ± 0.84	−0.048	(−0.367; 0.270)	0.759	0.843	−0.05
Medial (MJS)—Right	2.78 ± 1.01	2.91 ± 1.00	+0.130	(−0.080; 0.341)	0.216	0.721	0.22
Medial (MJS)—Left	2.68 ± 0.87	2.54 ± 0.81	−0.133	(−0.392; 0.125)	0.302	0.754	−0.18
Anterior (AJS)—Right	2.28 ± 0.65	2.44 ± 0.79	+0.164	(−0.040; 0.367)	0.112	0.558	0.28
Anterior (AJS)—Left	2.51 ± 0.83	2.46 ± 0.79	−0.048	(−0.335; 0.238)	0.733	0.843	−0.06
Posterior (PJS)—Right	2.96 ± 0.92	3.00 ± 1.19	+0.042	(−0.306; 0.391)	0.607	0.843	0.04
Posterior (PJS)—Left	2.73 ± 0.86	2.98 ± 1.06	+0.248	(−0.062; 0.559)	0.087	0.558	0.28
Central (CJS)—Right	2.99 ± 0.98	3.01 ± 1.18	+0.018	(−0.296; 0.332)	0.907	0.907	0.02
Central (CJS)—Left	2.98 ± 1.17	2.86 ± 1.14	−0.115	(−0.425; 0.195)	0.438	0.790	−0.13

T0 and T1 values are the mean ± SD; change = T1 − T0. A paired t test was used when differences were normally distributed and the Wilcoxon signed-rank test when Shapiro–Wilk *p* < 0.05 (right and left posterior, left central). dz = paired Cohen d. SD = standard deviation; CI = confidence interval; LJS, MJS, AJS, PJS, CJS = lateral, medial, anterior, posterior and central joint spaces. FDR = Benjamini–Hochberg across 10 tests. Bootstrap (10,000 resamples) and sign-permutation tests gave identical conclusions (Table S8).

**Table 4:** Equivalence testing of joint space changes (TOST, Δ = ±0.5 mm).

Joint Space	Change (mm)	90% CI (mm)	*p* (TOSTs)	Equivalent
Lateral (LJS)—Right	+0.103	(−0.138; 0.344)	0.004	Yes
Lateral (LJS)—Left	−0.048	(−0.314; 0.217)	0.004	Yes
Medial (MJS)—Right	+0.130	(−0.045; 0.305)	<0.001	Yes
Medial (MJS)—Left	−0.133	(−0.348; 0.082)	0.004	Yes
Anterior (AJS)—Right	+0.164	(−0.006; 0.333)	0.001	Yes
Anterior (AJS)—Left	−0.048	(−0.287; 0.190)	0.002	Yes
Posterior (PJS)—Right	+0.042	(−0.248; 0.332)	0.006	Yes
Posterior (PJS)—Left	+0.248	(−0.009; 0.506)	0.054	No
Central (CJS)—Right	+0.018	(−0.243; 0.279)	0.002	Yes
Central (CJS)—Left	−0.115	(−0.373; 0.143)	0.008	Yes

TOSTs = two one-sided tests. Equivalence was tested against a margin of ±0.5 mm; a result is equivalent when the 90% confidence interval lies entirely within that margin. CI = confidence interval; LJS, MJS, AJS, PJS, CJS = lateral, medial, anterior, posterior and central joint spaces.

**Table 5:** T0-to-T1 transition tables for Pullinger categories.

	**Right condyle**			
	T1 Posterior	T1 Concentric	T1 Anterior	Total
T0 Posterior	4	0	0	4
T0 Concentric	0	4	7	11
T0 Anterior	2	9	7	18
T1 Total	6	13	14	33
	**Left condyle**			
	T1 Posterior	T1 Concentric	T1 Anterior	Total
T0 Posterior	4	2	2	8
T0 Concentric	1	9	4	14
T0 Anterior	0	3	8	11
T1 Total	5	14	14	33

Categories: index < −12 posterior, −12 to +12 concentric, and > +12 anterior. Right: McNemar–Bowker χ^2^ = 2.250, df = 2, *p* = 0.325; unchanged in 15/33 (45.5%); weighted κ = 0.21. Left: McNemar–Bowker χ^2^ = 2.476, df = 3, *p* = 0.480; unchanged in 21/33 (63.6%); weighted κ = 0.47. Transitions were symmetric on both sides, with no systematic directional shift.

**Table 6:** Change in dental variables from T0 to T1 (*n* = 33).

Variable	T0	T1	Change	95% CI	*p*	dz
Intermolar width (mm)	42.91 ± 3.58	47.97 ± 3.37	+5.07	(4.21; 5.93)	<0.001	2.09
Overjet (mm)	1.97 ± 3.60	3.37 ± 2.64	+1.40	(0.61; 2.19)	0.001	0.63
Overbite (mm)	1.74 ± 2.16	0.36 ± 2.33	−1.38	(−2.16; −0.60)	0.001	−0.63

T0 and T1 values are mean ± SD; change = T1 − T0; CI = confidence interval; dz = paired Cohen d. All differences were normally distributed (Shapiro–Wilk *p* ≥ 0.280), and paired *t* tests were used. Bootstrap 95% CIs: intermolar width (4.26; 5.88), overjet (0.67; 2.16), and overbite (−2.12; −0.66), all consistent with parametric intervals.

## Data Availability

The data presented in this study are available on request from the corresponding author. The data are not publicly available because they consist of patient imaging records and their sharing is restricted by privacy and ethical considerations.

## Supplementary Materials

The following supporting information can be downloaded at https://www.mdpi.com/article/10.3390/jcm15218503/s1: Table S1: Between-group comparison of baseline (T0) values; Table S2: Number of patients exceeding predefined change thresholds; Table S3: Mixed-effects model results (sensitivity analysis); Table S4: Change in the Pullinger and coronal indices from T0 to T1 (*n* = 33); Table S5: Association between dental change and joint-space change (exploratory); Table S6: Effect of appliance type on change (adjusted for baseline value, age and interval; exploratory); Table S7: Effect of appliance type on dental change (adjusted for baseline value, age and interval; exploratory); Table S8: Bootstrap confidence intervals and permutation tests; Table S9: Right–left symmetry at each time point.

## Abbreviations

The following abbreviations are used in this manuscript:

AJS	Anterior joint space
CBCT	Cone-beam computed tomography
CJS	Central joint space
FDR	False discovery rate
ICC	Intraclass correlation coefficient
LJS	Lateral joint space
LoA	Limit of agreement
MARPE	Microimplant-assisted rapid palatal expansion
MJS	Medial joint space
PJS	Posterior joint space
RME	Rapid maxillary expansion
SDC	Smallest detectable change
SEM	Standard error of measurement
T0	Before expansion
T1	End of passive retention
TMJ	Temporomandibular joint
TOSTs	Two one-sided tests
