# Supplementary material for: The Condyle–Fossa Relationship After Maxillary Expansion with Tooth-Borne and Tooth-Bone-Borne Appliances: A Retrospective Cone-Beam Computed Tomography Study at the End of Retention
Source: J Clin Med. 2026 Nov 2;15(21):8503. doi: 10.3390/jcm15218503 (PMC13763922; doi:10.3390/jcm15218503)
Supplement: Supplementary file 1 [file jcm-15-08503-s001.zip › jcm-4604885-supplementary.pdf]

Supplementary Materials

The Condyle–Fossa Relationship After Maxillary Expansion with Tooth-Borne and Tooth-Bone-Borne Appliances: A Retrospective Cone-Beam Computed Tomography Study at the End of Retention

Table S1. Between-group comparison of baseline (T0) values

Variable	Hyrax (n = 16)	MARPE (n = 17)	*p*	*p* (FDR)
Lateral (LJS) – Right	3.12 ± 1.28	3.08 ± 1.22	0.971	0.971
Lateral (LJS) – Left	2.91 ± 0.91	3.20 ± 1.30	0.639	0.922
Medial (MJS) – Right	2.17 ± 0.68	3.35 ± 0.94	<0.001	0.011
Medial (MJS) – Left	2.63 ± 0.84	2.72 ± 0.91	0.759	0.922
Anterior (AJS) – Right	2.25 ± 0.47	2.31 ± 0.80	0.842	0.955
Anterior (AJS) – Left	2.39 ± 0.61	2.62 ± 1.00	0.678	0.922
Posterior (PJS) – Right	2.96 ± 1.07	2.96 ± 0.79	0.613	0.922
Posterior (PJS) – Left	2.72 ± 0.98	2.74 ± 0.76	0.759	0.922
Central (CJS) – Right	2.88 ± 0.87	3.11 ± 1.09	0.377	0.922
Central (CJS) – Left	3.13 ± 0.93	2.83 ± 1.37	0.482	0.922
Pullinger index (%) – Right	11.85 ± 15.51	12.69 ± 17.02	0.577	0.922
Pullinger index (%) – Left	5.64 ± 19.53	3.59 ± 21.58	0.914	0.971
Coronal index (%) – Right	16.82 ± 17.34	−5.40 ± 19.67	0.004	0.038
Coronal index (%) – Left	5.06 ± 13.32	6.48 ± 21.76	0.759	0.922
Intermolar width (mm)	43.28 ± 4.03	42.56 ± 3.18	0.482	0.922
Overjet (mm)	0.72 ± 3.60	3.15 ± 3.26	0.040	0.227
Overbite (mm)	2.48 ± 2.13	1.05 ± 2.02	0.058	0.248

Values are mean ± SD; Mann–Whitney *U* test. FDR = false discovery rate (Benjamini–Hochberg correction across 17 tests). SD = standard deviation; MARPE = microimplant-assisted rapid palatal expansion; LJS, MJS, AJS, PJS, CJS = lateral, medial, anterior, posterior and central joint spaces.

Table S2. Number of patients exceeding predefined change thresholds

Joint space	$\lvert\Delta\rvert$ > 0.5 mm	$\lvert\Delta\rvert$ > 1.0 mm	$\lvert\Delta\rvert$ > 1.5 mm
Lateral (LJS) – Right	14 (42)	8 (24)	3 (9)
Lateral (LJS) – Left	16 (48)	5 (15)	2 (6)

Joint space	|Δ| > 0.5 mm	|Δ| > 1.0 mm	|Δ| > 1.5 mm
Medial (MJS) – Right	11 (33)	3 (9)	0 (0)
Medial (MJS) – Left	11 (33)	5 (15)	2 (6)
Anterior (AJS) – Right	12 (36)	2 (6)	0 (0)
Anterior (AJS) – Left	18 (55)	7 (21)	2 (6)
Posterior (PJS) – Right	12 (36)	7 (21)	4 (12)
Posterior (PJS) – Left	13 (39)	5 (15)	2 (6)
Central (CJS) – Right	19 (58)	5 (15)	3 (9)
Central (CJS) – Left	12 (36)	6 (18)	5 (15)

Values are n (%) of 33 patients. Δ = T1 − T0. The smallest detectable change for an individual T0–T1 change ranged from 0.28 to 0.46 mm (Table 2), so every change counted in this table exceeded measurement error.

Table S3. Mixed-effects model results (sensitivity analysis)

Variable	Time effect (T1 − T0)	SE	95% CI	*p*	*p* (FDR)	Time × side *p*	Patient ICC
Lateral (LJS)	+0.027	0.105	(−0.179; 0.234)	0.796	0.985	0.468	0.38
Medial (MJS)	−0.002	0.083	(−0.164; 0.161)	0.985	0.985	0.105	0.35
Anterior (AJS)	+0.058	0.087	(−0.112; 0.227)	0.506	0.985	0.215	0.37
Posterior (PJS)	+0.145	0.114	(−0.079; 0.370)	0.204	0.985	0.363	0.56
Central (CJS)	−0.048	0.108	(−0.260; 0.163)	0.653	0.985	0.533	0.61
Pullinger index (%)	+0.414	2.531	(−4.546; 5.375)	0.870	0.985	0.126	0.25
Coronal index (%)	+0.570	2.425	(−4.182; 5.323)	0.814	0.985	0.270	0.00

Model: measurement ~ time + (1|patient) + (1|patient:side). Right and left condyles were analysed in a single model accounting for within-patient dependence. The time × side interaction was tested by likelihood-ratio test. ICC = between-patient variance as a proportion of total variance. SE = standard error; CI = confidence interval; FDR = false discovery rate.

Table S4. Change in the Pullinger and coronal indices from T0 to T1 (n = 33)

Index	T0 (%)	T1 (%)	Change (points)	95% CI	*p*	*p* (FDR)	dz
Pullinger index (%) – Right	12.28 ± 16.05	8.89 ± 20.91	−3.39	(−10.85; 4.07)	0.361	0.508	−0.16
Pullinger index (%) – Left	4.58 ± 20.32	8.80 ± 21.06	+4.22	(−2.75; 11.19)	0.226	0.508	0.21
Coronal index (%) – Right	5.38 ± 21.48	3.30 ± 19.87	−2.07	(−8.63; 4.49)	0.524	0.524	−0.11

Index	T0 (%)	T1 (%)	Change (points)	95% CI	*p*	*p* (FDR)	dz
Coronal index (%) – Left	5.79 ± 17.90	9.00 ± 17.11	+3.21	(−4.16; 10.58)	0.381	0.508	0.15

Values are mean ± SD. Pullinger index = (PJS − AJS)/(PJS + AJS) × 100; coronal index = (LJS − MJS)/(LJS + MJS) × 100. Shapiro–Wilk $p > 0.05$ for all differences, so paired *t* tests were used. dz = paired Cohen d; CI = confidence interval; FDR = false discovery rate.

Table S5. Association between dental change and joint-space change (exploratory)

Predictor	Outcome	*r* (Pearson)	95% CI	*p*	*p* (FDR)	ϱ (Spearman)
Intermolar width Δ	Lateral (LJS) – Right	+0.109	(−0.24; 0.44)	0.545	0.895	+0.081
Intermolar width Δ	Lateral (LJS) – Left	+0.157	(−0.20; 0.47)	0.383	0.895	+0.221
Intermolar width Δ	Medial (MJS) – Right	+0.042	(−0.31; 0.38)	0.817	0.895	+0.035
Intermolar width Δ	Medial (MJS) – Left	+0.102	(−0.25; 0.43)	0.571	0.895	+0.159
Intermolar width Δ	Anterior (AJS) – Right	−0.041	(−0.38; 0.31)	0.821	0.895	−0.047
Intermolar width Δ	Anterior (AJS) – Left	−0.155	(−0.47; 0.20)	0.389	0.895	−0.167
Intermolar width Δ	Posterior (PJS) – Right	+0.096	(−0.26; 0.43)	0.594	0.895	+0.037
Intermolar width Δ	Posterior (PJS) – Left	+0.005	(−0.34; 0.35)	0.977	0.977	+0.153
Intermolar width Δ	Central (CJS) – Right	+0.194	(−0.16; 0.50)	0.280	0.895	+0.143
Intermolar width Δ	Central (CJS) – Left	−0.055	(−0.39; 0.29)	0.763	0.895	−0.081
Intermolar width Δ	Pullinger index – Right	+0.086	(−0.27; 0.42)	0.634	0.895	+0.134
Intermolar width Δ	Pullinger index – Left	+0.155	(−0.20; 0.47)	0.388	0.895	+0.125
Overjet Δ	Lateral (LJS) – Right	−0.280	(−0.57; 0.07)	0.114	0.868	−0.195
Overjet Δ	Lateral (LJS) – Left	−0.047	(−0.38; 0.30)	0.795	0.868	−0.222
Overjet Δ	Medial (MJS) – Right	−0.138	(−0.46; 0.22)	0.443	0.868	−0.180
Overjet Δ	Medial (MJS) – Left	+0.069	(−0.28; 0.40)	0.703	0.868	+0.068
Overjet Δ	Anterior (AJS) – Right	−0.135	(−0.46; 0.22)	0.452	0.868	−0.232
Overjet Δ	Anterior (AJS) – Left	+0.139	(−0.21; 0.46)	0.440	0.868	+0.175
Overjet Δ	Posterior (PJS) – Right	−0.051	(−0.39; 0.30)	0.778	0.868	+0.168
Overjet Δ	Posterior (PJS) – Left	−0.004	(−0.35; 0.34)	0.982	0.982	+0.027
Overjet Δ	Central (CJS) – Right	−0.241	(−0.54; 0.11)	0.176	0.868	−0.214
Overjet Δ	Central (CJS) – Left	−0.071	(−0.40; 0.28)	0.694	0.868	−0.064
Overjet Δ	Pullinger index – Right	+0.108	(−0.24; 0.44)	0.550	0.868	+0.262

Predictor	Outcome	*r* (Pearson)	95% CI	*p*	*p* (FDR)	ϱ (Spearman)
Overjet Δ	Pullinger index – Left	−0.082	(−0.41; 0.27)	0.650	0.868	+0.002
Overbite Δ	Lateral (LJS) – Right	+0.125	(−0.23; 0.45)	0.487	0.585	+0.009
Overbite Δ	Lateral (LJS) – Left	+0.179	(−0.18; 0.49)	0.319	0.585	+0.281
Overbite Δ	Medial (MJS) – Right	+0.129	(−0.22; 0.45)	0.476	0.585	+0.136
Overbite Δ	Medial (MJS) – Left	+0.133	(−0.22; 0.46)	0.459	0.585	+0.171
Overbite Δ	Anterior (AJS) – Right	+0.083	(−0.27; 0.41)	0.646	0.704	+0.106
Overbite Δ	Anterior (AJS) – Left	+0.225	(−0.13; 0.53)	0.209	0.585	+0.255
Overbite Δ	Posterior (PJS) – Right	−0.177	(−0.49; 0.18)	0.324	0.585	−0.130
Overbite Δ	Posterior (PJS) – Left	−0.154	(−0.47; 0.20)	0.391	0.585	−0.041
Overbite Δ	Central (CJS) – Right	−0.129	(−0.45; 0.22)	0.474	0.585	−0.196
Overbite Δ	Central (CJS) – Left	+0.052	(−0.30; 0.39)	0.773	0.773	+0.055
Overbite Δ	Pullinger index – Right	−0.143	(−0.46; 0.21)	0.427	0.585	−0.185
Overbite Δ	Pullinger index – Left	−0.270	(−0.56; 0.08)	0.128	0.585	−0.229

Δ = T1 − T0. FDR correction (Benjamini–Hochberg) was applied across the 12 tests per predictor. No association reached nominal significance. CI = confidence interval (Fisher *z*); FDR = false discovery rate.

Table S6. Effect of appliance type on change (adjusted for baseline value, age and interval; exploratory)

Outcome	Hyrax Δ	MARPE Δ	Adjusted difference	95% CI	*p*	*p* (FDR)	*p* (permutation)
Lateral (LJS) – Right	−0.238	+0.424	+0.370	(−0.25; 0.99)	0.229	0.946	0.173
Lateral (LJS) – Left	−0.088	−0.012	+0.187	(−0.40; 0.77)	0.519	0.946	0.477
Medial (MJS) – Right	+0.212	+0.053	+0.135	(−0.45; 0.72)	0.639	0.946	0.535
Medial (MJS) – Left	−0.125	−0.141	−0.087	(−0.61; 0.44)	0.738	0.946	0.716
Anterior (AJS) – Right	+0.144	+0.182	+0.101	(−0.40; 0.60)	0.684	0.946	0.647
Anterior (AJS) – Left	−0.106	+0.006	+0.370	(−0.21; 0.95)	0.199	0.946	0.151
Posterior (PJS) – Right	−0.169	+0.241	+0.198	(−0.62; 1.01)	0.623	0.946	0.583
Posterior (PJS) – Left	+0.225	+0.271	+0.249	(−0.49; 0.98)	0.494	0.946	0.446
Central (CJS) – Right	−0.094	+0.124	+0.064	(−0.69; 0.82)	0.863	0.946	0.841
Central (CJS) – Left	−0.200	−0.035	+0.102	(−0.57; 0.78)	0.759	0.946	0.735
Pullinger index – Right	−4.898	−1.973	+0.567	(−16.36; 17.49)	0.946	0.946	NA

Outcome	Hyrax Δ	MARPE Δ	Adjusted difference	95% CI	*p*	*p* (FDR)	*p* (permutation)
Pullinger index – Left	+5.072	+3.419	−0.658	(−16.23; 14.91)	0.932	0.946	NA
Coronal index – Right	−10.479	+5.840	+5.609	(−9.40; 20.62)	0.450	0.946	NA
Coronal index – Left	+1.811	+4.532	+6.152	(−7.35; 19.65)	0.359	0.946	NA

Model: Δ ~ appliance + baseline (T0) value + age + T0–T1 interval (analysis of covariance). The adjusted difference is MARPE − Hyrax. FDR across 14 tests. Permutation *p*-values are based on 10,000 permutations of appliance labels (NA = not performed for the indices). Appliance type was not randomly allocated; associations are exploratory. CI = confidence interval; FDR = false discovery rate.

Table S7. Effect of appliance type on dental change (adjusted for baseline value, age and interval; exploratory)

Outcome	Hyrax Δ	MARPE Δ	Adjusted difference	95% CI	*p*
Intermolar width Δ (mm)	+5.25	+4.89	−1.25	(−3.12; 0.63)	0.185
Overjet Δ (mm)	+2.26	+0.59	−0.19	(−1.57; 1.19)	0.781
Overbite Δ (mm)	−1.29	−1.46	−1.19	(−3.04; 0.65)	0.196

Model: Δ ~ appliance + baseline (T0) value + age + T0–T1 interval. Adjusted difference = MARPE − Hyrax. CI = confidence interval.

Table S8. Bootstrap confidence intervals and permutation tests

Variable	Change	Bootstrap 95% CI	*p* (permutation)
Lateral (LJS) – Right	+0.103	(−0.170; 0.376)	0.468
Lateral (LJS) – Left	−0.048	(−0.358; 0.245)	0.760
Medial (MJS) – Right	+0.130	(−0.067; 0.333)	0.209
Medial (MJS) – Left	−0.133	(−0.367; 0.115)	0.320
Anterior (AJS) – Right	+0.164	(−0.033; 0.355)	0.109
Anterior (AJS) – Left	−0.048	(−0.321; 0.221)	0.728
Posterior (PJS) – Right	+0.042	(−0.270; 0.400)	0.825
Posterior (PJS) – Left	+0.248	(−0.036; 0.555)	0.123
Central (CJS) – Right	+0.018	(−0.276; 0.321)	0.922
Central (CJS) – Left	−0.115	(−0.415; 0.179)	0.456
Pullinger index – Right	−3.391	(−10.502; 3.626)	0.358
Pullinger index – Left	+4.220	(−2.238; 10.971)	0.235
Intermolar width	+5.067	(4.258; 5.876)	<0.001
Overjet	+1.400	(0.667; 2.158)	<0.001

**Variable**	**Change**	**Bootstrap 95% CI**	***p* (permutation)**
Overbite	−1.379	(−2.121; −0.661)	0.001

Based on 10,000 bootstrap resamples (percentile intervals) and 10,000 sign permutations. CI = confidence interval.

Table S9. Right–left symmetry at each time point

**Variable**	**Time**	**Right**	**Left**	**Difference**	***p***	***p* (FDR)**
Lateral (LJS)	T0	3.10	3.06	+0.04	0.640	0.896
Lateral (LJS)	T1	3.20	3.01	+0.19	0.382	0.764
Medial (MJS)	T0	2.78	2.68	+0.10	0.572	0.896
Medial (MJS)	T1	2.91	2.54	+0.37	0.057	0.447
Anterior (AJS)	T0	2.28	2.51	−0.23	0.158	0.554
Anterior (AJS)	T1	2.44	2.46	−0.02	0.903	0.982
Posterior (PJS)	T0	2.96	2.73	+0.22	0.155	0.554
Posterior (PJS)	T1	3.00	2.98	+0.02	0.599	0.896
Central (CJS)	T0	2.99	2.98	+0.02	0.915	0.982
Central (CJS)	T1	3.01	2.86	+0.15	0.349	0.764
Pullinger index	T0	12.28	4.58	+7.70	0.064	0.447
Pullinger index	T1	8.89	8.80	+0.09	0.982	0.982
Coronal index	T0	5.38	5.79	−0.41	0.936	0.982
Coronal index	T1	3.30	9.00	−5.70	0.235	0.658

Values are means (mm for joint spaces, % for indices). Paired *t* test, or Wilcoxon signed-rank test when the differences were not normally distributed. FDR = false discovery rate (Benjamini–Hochberg).
